# Case of Reproduction of Tooth after Gun-Shot Wound of Maxillary Bone

**Published:** 1868-06

**Authors:** James S. Blain

**Affiliations:** Brunswick, Georgia


### Selections.

---

CASE OF REPRODUCTION OF TOOTH AFTER GUN-SHOT WOUND OF MAXILLARY BONE.—BY JAMES S. BLAIN, M. D., OF GEORGIA.—As it is the duty of each member of our profession to contribute as much as possible to the advancement of medical knowledge and science, and to give publicity to any facts, either new or strange, which may come under their observation, I propose to report a case, which though it may be of no great practical benefit to the profession, may still be of interest, as illustrating the wondrous reparative power of nature.

Similar cases may have been observed by some of the surgeons connected with the armies of the late Confederacy, though I have seen no report made of them. It was my fortune to wield the sword during our recent struggle; for this reason my surgical experience was very limited.

I have no notes of the following case, and in consequence, will necessarily be compelled to avoid detail.

Lieut. E. W. Blount, of the 26th Reg't Ga., Vols., Gordon's Brigade, was wounded, if I mistake not, at the battle of Spottsylvania Court House, in 1864. A Minie ball passed in at one side of his face and out of the other, fracturing the superior maxillary bone on the left side and the inferior on the right, and nearly severing the tongue. When operated upon, a portion of the maxillary bone, including the second bi-cuspid tooth, was resected. His recovery was, for a long time, extremely doubtful; but, by kind treatment and a naturally robust constitution, he was finally restored to health. A few days since he called at my office to see me, and, upon examination, I found that cartilagious union had taken place between the divided ends of bone, and that, in this cartilage, *nature* had *reproduced* the tooth which, as above mentioned, was removed with the resected portion of bone. Lieut. B. is now in the enjoyment of good health, and though the motion of the inferior maxillary is very imperfect, still it is sufficient for the performance of the functions of life.

If you think the above case of sufficient interest to merit publication, please give it a place in your journal; if not, deal with it as you think proper.

Very respectfully, you obedient servant,

JAMES S. BLAIN, M. D.

BRUNSWICK, GA., Aug., 10th, 1866.

